# Coronary Arteriovenous Fistula Causing Hydrops Fetalis

**DOI:** 10.1155/2014/487281

**Published:** 2014-08-26

**Authors:** Nilüfer Çetiner, Sinem Altunyuva Usta, Figen Akalın

**Affiliations:** Department of Pediatric Cardiology, Marmara University, Mimar Sinan Caddesi, No. 41, Pendik, 34890 İstanbul, Turkey

## Abstract

Fetal heart failure and hydrops fetalis may occur due to systemic arteriovenous fistula because of increased cardiac output. Arteriovenous fistula of the central nervous system, liver, bone or vascular tumors such as sacrococcygeal teratoma were previously reported to be causes of intrauterine heart failure. However, coronary arteriovenous fistula was not reported as a cause of fetal heart failure previously. It is a rare pathology comprising 0.2–0.4% of all congenital heart diseases even during postnatal life. Some may remain asymptomatic for many years and diagnosed by auscultation of a continuous murmur during a routine examination, while a larger fistulous coronary artery opening to a low pressure cardiac chamber may cause ischemia of the affected myocardial region due to steal phenomenon and may present with cardiomyopathy or congestive heart failure during childhood. We herein report a neonate with coronary arteriovenous fistula between the left main coronary artery and the right ventricular apex, who presented with hydrops fetalis during the third trimester of pregnancy.

## 1. Introduction

Congestive heart failure and hydrops fetalis may occur due to cardiac or noncardiac causes during intrauterine life. About 26% of the fetuses with hydrops fetalis have cardiac pathologises and 41% of them are congenital heart diseases [1]. Coronary AV fistula (CAVF) is a rare congenital heart disease even during postnatal life with an overall incidence of 1/50000 [2]. They constitute 0.2 to 0.4% of all congenital heart diseases and more than half of all congenital coronary anomalies [3]. Systemic arteriovenous fistula is known to cause high-output heart failure and hydrops fetalis during intrauterine life. However, CAVF was not reported as a cause of intrauterine cardiac failure or hydrops fetalis previously. Some patients with CAVF may remain asymptomatic for many years unless the amount of shunt through AV fistula is not large and symptoms usually occur in older age; many patients are diagnosed due to auscultation of a continuous murmur during routine examination [4, 5]. On the other hand, larger fistula may cause congestive heart failure due to increased volume load of the left ventricle or myocardial ischemia, cardiomyopathy, or myocardial infarction may occur due to steal phenomenon during infancy or childhood. Intrauterine diagnosis of CAVF was not also reported previously.

We herein present a case with CAVF leading to congestive heart failure and hydrops fetalis during intrauterine life.

## 2. Case Report

**Figure 1:**
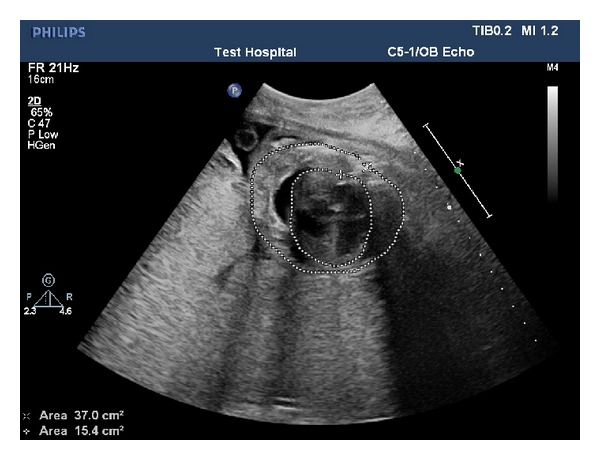
Four-chamber view of the fetal heart with increased cardiothoracic ratio.

**Figure 2:**
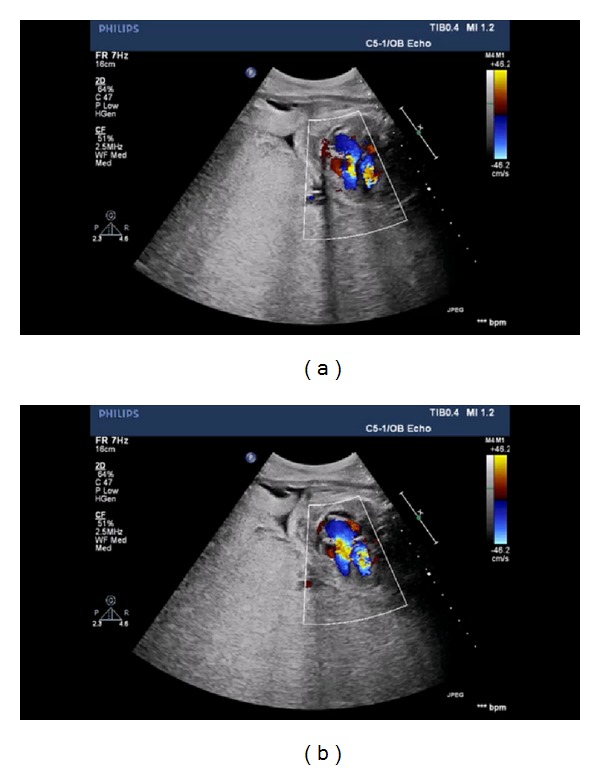
Pulsed wave Doppler examination demonstrating significant tricuspid regurgitation.

**Figure 3:**
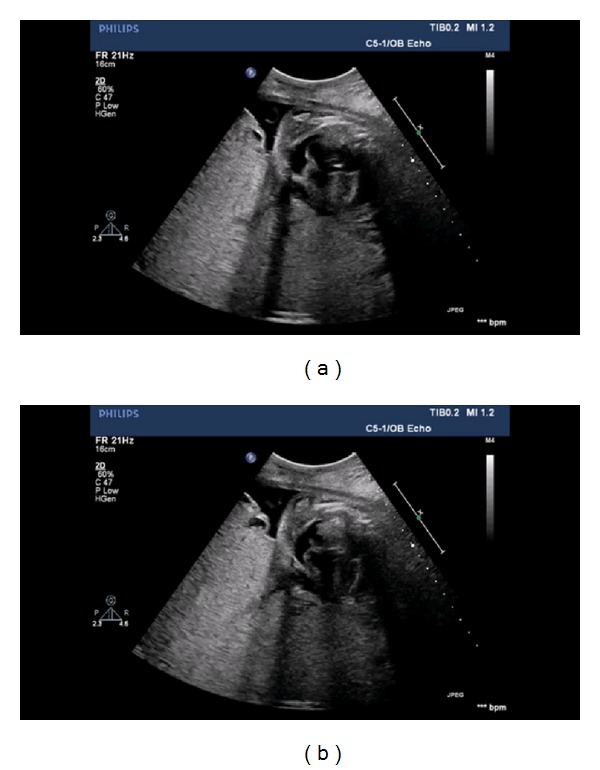
Fetal echocardiographic appearance of dilated coronary sinus.

**Figure 4:**
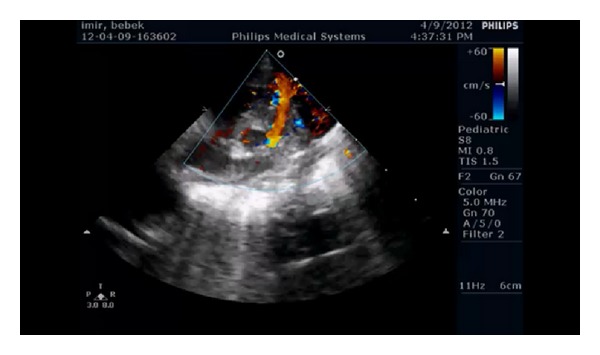
Postnatal echocardiographic examination demonstrating the large arteriovenous fistula connecting the left coronary artery with the apex of the right ventricle.

A 22-year-old lady at the 26th week of pregnancy was referred to our clinic for fetal echocardiography; the indication was pericardial effusion detected during routine obstetric ultrasonography. She was previously healthy and this was her first pregnancy; her medical history was uneventful; however, her obstetric follow-up was not regular. Fetal echocardiography revealed pericardial, pleural, abdominal free fluid and skin edema. Intracardiac anatomy seemed to be normal despite poor echocardiographic images. However, cardiothoracic ratio was increased (heart was 1/2 times as large as the thoracic cavity), and significant holosystolic tricuspid regurgitation was observed; coronary sinus was found to be dilated (Figures 1, 2, and 3). Severe bradycardia was present during the entire examination. Since these findings suggested hydrops fetalis and severe fetal heart failure, the patient was immediately referred to obstetrics and gynecology clinic where an emergency cesarian section was performed. She gave birth to a premature boy weighing 1050 grams with no spontaneous respiration, his heart rate was below 60/minute, and he was hypotonic. Based on these findings an emergency cardiopulmonary resuscitation was started; the patient was intubated and admitted to the neonatal intensive care unit. His skin was pale, cold, and a generalized edema was present. Lung auscultation revealed bilateral equal aeration and fine rales. Heart sounds were muffled but rhythmic and bradycardic with a rate of 100 bpm without any murmur. Peripheral pulses were weak; blood pressure could not be measured. During abdominal examination, the liver was 3 cm palpable and spleen was not palpated. Heart failure treatment was started under the support of mechanical ventilation. Blood gas analysis showed severe metabolic acidosis. Medication included dopamine and dobutamine infusion as inotropic agents. Transthoracic echocardiography was performed in order to identify the cause of congestive heart failure. A large arteriovenous fistula connecting the left coronary artery with the apex of the right ventricle was found (Figure 4); left ventricular contractility was acceptable; and other structures of the heart were normal. No intervention was possible because of the poor clinical condition of the baby. Correction of the acidosis and maintaining the blood pressure were not possible and the patient was lost at the 4th hour of life in spite of aggressive inotropic and ventilatory support.

## 3. Discussion

High-output congestive heart failure leading to hydrops fetalis may occur due to systemic arteriovenous fistula. Intrauterine heart failure and hydrops fetalis due to CAVF were not reported previously; however, systemic arteriovenous fistula involving other sites such as central nervous system, liver, bone or vascular tumors such as sacrococcygeal teratoma are known but rare causes of fetal heart failure [6, 7]. Arteriovenous malformations cause volume overload to the ventricles. In fetal heart, right ventricle provides two-thirds of the total cardiac output. Abnormal loading conditions are not tolerated by the fetal heart as the mature heart because of limited circulatory reserves. Resting heart rate is high and cardiac output cannot be increased by increasing heart rate; fetal myocardium is less compliant and generates less contractile force because of different structure and metabolism of the contractile apparatus comparing the mature heart. Any cardiac insult to the fetus causes brain sparing circulatory changes, abnormal remodeling of the ventricles, decreased renal perfusion, activation of renin-angiotensin system, salt and water retention, atrial natriuretic peptide release, vascular smooth muscle relaxation, and increased capillary permeability. Extracellular fluid clearance depends on high-rate lymph drainage in the fetus and even moderate increases in systemic venous pressure cause fluid accumulation in pleural, pericardial, and abdominal spaces and skin, which are the manifestations of hydrops fetalis. In our case, hydrops fetalis has developed due to severe heart failure caused by coronary arteriovenous fistula.

On the other hand in many fetuses with nonimmune hydrops fetalis the real cause may not be identified; early echocardiographic examination or autopsy studies may be helpful in defining such abnormalities and the diagnosis may be difficult if they are not looked for. Common congenital heart diseases of postnatal life are usually tolerated during intrauterine life and do not cause intrauterine hydrops; however, intrauterine rhythm abnormalities, intrauterine infections, or congenital heart diseases associated with genetic abnormalities, structural malformations (Ebstein's anomaly and premature closure of the foramen ovale), myocardiopathy, vascular obstruction (tumor, structural abnormalities, and fibroelastosis), vascular malformation, and hemangioma are other possible cardiac causes [8, 9]. Since it is difficult to detect pathologies such as coronary arteriovenous fistula in utero, real incidence among the cases with idiopathic nonimmune hydrops fetalis may be higher. The dilated appearance of the coronary sinus may be actually the appearance of dilated coronary artery because of CVAF in our case.

Fetal heart failure or hydrops fetalis usually does not cause any symptom in mother and is detected during routine obstetric ultrasonography, which was the case in our patient. Transplacental treatment of the fetal heart failure by giving inotropic agents to mother may be considered; however, once hydrops fetalis develops, the only option is the emergency delivery of the baby; we also proceeded in the same way.

Congenital coronary artery fistulas are defined as direct links between the coronary arteries and any of the four cardiac chambers. The dilated coronary arteries have no capillary system and rarely open into the coronary sinus, superior vena cava, pulmonary artery, or pulmonary vein [10, 11]. Clinical presentation and complications depend on the size of the fistula and amount of shunt. According to Fernandes et al. 73% of isolated coronary AV fistulas remain asymptomatic for many years and symptoms usually occur in older age; dyspnea and congestive heart failure are the presenting symptoms in 75% of the patients over the age of 40 [1, 12–14]. Auscultation of the typical continuous murmur suggesting a patent ductus arteriosus during a routine physical examination may be the mode of presentation during childhood [4]. In patients with large left to right shunt, congestive heart failure may be seen in infancy [15]. Endocarditis, myocardial ischemia, high-output cardiac failure, thrombosis, and sudden death are other complications of the disease [1].

Coronary AV fistula originates from the right coronary artery in 55–60% of the cases and 90% of them open into the right side of the heart (45% into the right ventricle, 25% into the right atrium, 15–20% into the pulmonary artery, and 7% into the coronary sinus). In Lowe and Sabiston's series of 286 cases, right coronary artery was involved in 56%, left coronary artery was involved in 36%, and both coronary arteries were involved in 5% of the patients. The fistula opened into the right ventricle in 39%, right atrium in 33%, pulmonary artery in 20%, left atrium in 6%, and left ventricle in 2% of the cases [15]. In our case, coronary artery fistula originated from the left coronary artery and was draining into the right ventricular apex.

Although the cardiac catheterization and coronary angiography are required for demonstration of the exact anatomy of the fistula and diagnosis of the additional cardiac anomalies, in recent years, technical developments in noninvasive diagnostic methods such as 2D and Doppler echocardiography provide sufficient information in CAVF. In our case, CAVF was diagnosed within the first hour of life by transthoracic echocardiography [16].

Treatment options are surgical or transcatheter closure of the fistula. Angiography was planned; however, the clinical condition of the patient was not suitable for any kind of intervention and he could stay alive for only 4 hours.

In conclusion, coronary arteriovenous fistula is a rare congenital heart disease which may cause intrauterine heart failure and must be considered in differential diagnosis of the fetuses with nonimmune hydrops fetalis. Presence of hydrops fetalis in routine obstetric ultrasonography is an indication for fetal echocardiography.

## Supplementary Material

3 of these images belong to fetus who is in the 26th week of gestation and one of these images belongs to same patient after birth.
Figure 1: Four-chamber view of the fetal heart with increased cardiothoracic ratio.
Figure 2: Pulsed wave Doppler examination demonstrating significant tricuspid regurgitation.
Figure 3: Fetal echocardiographic appearance of dilated coronary sinus.
Figure 4: Postnatal echocardiographic examination demonstrating the large arteriovenous fistula connecting the left coronary artery with the apex of the right ventricle.
