# Supplementary figures and images for: Coronary Arteriovenous Fistula Causing Hydrops Fetalis
Source: Case Rep Obstet Gynecol. 2014 Aug 26;2014:487281. doi: 10.1155/2014/487281 (PMC4160637; doi:10.1155/2014/487281)

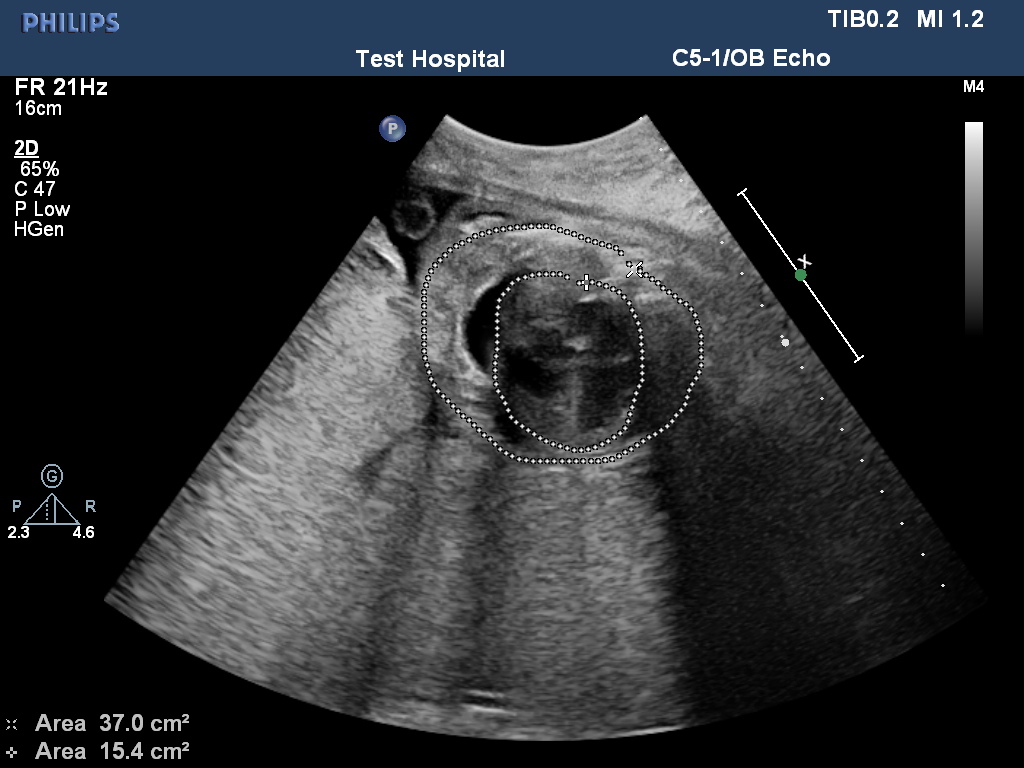

Supplement: Supplementary file 1 — 3 of these images belong to fetus who is in the 26th week of gestation and one of these images belongs to same patient after birth. Figure 1: Four-chamber view of the fetal heart with increased cardiothoracic ratio. Figure 2: Pulsed wave Doppler examination demonstrating significant tricuspid regurgitation. Figure 3: Fetal echocardiographic appearance of dilated coronary sinus. Figure 4: Postnatal echocardiographic examination demonstrating the large arteriovenous fistula connecting the left coronary artery with the apex of the right ventricle. [file 487281.f1.zip › Cardio-thoracic ratio.jpg]
